# On the temporality of creative insight: a psychological and phenomenological perspective

**DOI:** 10.3389/fpsyg.2014.01184

**Published:** 2014-10-17

**Authors:** Diego Cosmelli, David D. Preiss

**Affiliations:** Escuela de Psicología, Facultad de Ciencias Sociales, Pontificia Universidad Católica de Chile, SantiagoChile

**Keywords:** creativity, insight, flow of experience, temporality, socio-cultural approach

## Abstract

Research into creative insight has had a strong emphasis on the psychological processes underlying problem-solving situations as a standard model for the empirical study of this phenomenon. Although this model has produced significant advances in our scientific understanding of the nature of insight, we believe that a full comprehension of insight requires complementing cognitive and neuroscientific studies with a descriptive, first-person, phenomenological approach into how creative insight is experienced. Here we propose to take such first-person perspective while paying special attention to the temporal aspects of this experience. When this first-person perspective is taken into account, a dynamic past–future interplay can be identified at the core of the experience of creative insight, a structure that is compatible with both biological and biographical evidences. We believe this approach could complement and help bring together biological and psychological perspectives. Furthermore, we argue that because of its spontaneous but recurrent nature, creative insight could represent a relevant target for the phenomenological investigation of the flow of experience itself.

## INTRODUCTION

Try to recall the last time you had the experience of a sudden breakthrough in thinking about some unresolved issue. It can be as academic, as artistic, or as domestic as you please. This experience is usually accompanied by a more perceptible, sudden experience of resolution, which is called the insight into the problem’s nature (the Aha! experience). Try to recall the feeling of insight itself, which is sometimes related to a positive feeling that goes beyond the cognitive restructuring of the problem. Would you agree that, in some sense, the moment of creative insight involves an embodied expression of surprise and familiarity? Of something that is at the same time both new (future oriented) and already known (past oriented)?

The field of insight and creativity research is extremely active (see Runco, 2004; Hennessey and Amabile, 2010; Sawyer, 2012; Kounios and Beeman, 2014; for comprehensive reviews). Although we place ourselves within the general socio-cultural approach, here we will focus on one particular aspect of creativity, namely the experience of creative insight (Sternberg and Davidson, 1995). Despite the extensive development of research on the psychological, cognitive, neuroscientific, and social aspects of creativity, it is worth noting that a more descriptive, phenomenological approach to how creative insight is experienced is still quite limited (Bindeman, 1998; Nelson, 2005). Here, we will argue that, when such phenomenological approach is taken into account, the temporal nuances of the experience of insight are brought to the fore in a way that has consequences that transcend the study of creativity. These temporal nuances highlight the past-oriented and future-looking dimensions of insight. Interestingly, this temporal structure resembles biological dynamics that span from brain activity to evolution. Moreover, attention to the phenomenological structure of insight could help understand the connections between neurobiological accounts stemming from cognitive neuroscience and more biographical and psychological accounts collected by sociocultural and systems approaches to creativity (Sawyer, 2012).

## PSYCHOLOGICAL STUDIES OF CREATIVITY AND INSIGHT

It was not until Guilford’s (1950) APA address that the notion of creativity drew the attention of psychologists as a matter of scientific interest (Sternberg and Lubart, 1999; Sternberg and Grigorenko, 2001). Guilford also inspired the study of creativity in everyday situations by using psychometric methods. Since, divergent thinking tests, such as the Torrance Tests of Creative Thinking (Torrance, 1972), which were based on work by Guilford (1967), have been extensively used. These tests show participants questions that can be answered in an open way: for example, “List things that would happen if we lived in a world without gravity.” Many of these tests are based on verbal stimuli, although they can also be figural and involve, for example, the completion of a drawing. The answers are scored in dimensions such as fluency (how many responses are produced) and originality (the responses’ uniqueness), among others. Despite their influence, divergent thinking tests have been accused of trivializing the concept of “creativity,” for reasons we agree with (Sternberg and Lubart, 1999; Sternberg and Grigorenko, 2001). Specifically, the divergent thinking construct reduces the concept of creativity to a problem solving process that is restricted to a specific task and moment in time. Yet, as illustrated by both descriptive evidence about the creative process (Bindeman, 1998) and biographical analysis through case studies (Gruber and Wallace, 1999) or in-depth interviews (Csikszentmihalyi, 1996), the generation of a creative product in a relevant cultural domain is neither an instantaneous event nor simply a matter of purely reflective problem solving. It is well known that the acquisition and mastery of specialized knowledge of the domain, involving a sophisticated interplay between convergent and divergent psychological processes (Goel, 2014) are necessary to account for real-world creative activity. A case in point is that of Darwin, who, as documented by Gruber and Wallace (1999), developed four or five stages of his theory of evolution from 1831 to 1838, as demonstrated by the record of his observations and his thinking, which includes both verbal and visual graphics. The analyses of cases such as that of Darwin illustrate that “it would be difficult if not impossible to construct the narrative of a case study using only one timescale. Short-term activities and experiences are embedded within longer episodes, and so on” (Gruber and Wallace, 1999, p. 104).

Contrasting with the more encompassing problem of creativity as such, research on the more temporally restricted phenomenon of insight as an event has a longer history, extending back to the contributions of the Gestalt movement. It is interesting to note that whereas in its origins the concept of insight was related to the observation of problem solving behavior in open-ended situations—such as those observed by Kohler (1925) with chimpanzees—by the end of the 20th century it was mostly assessed in experimental contexts through closed-ended problems. Thus, creative insight became mainly conceptualized as a specific type of problem-solving process, specifically, one that is not lived incrementally but is rather characterized by an impasse and a sudden, abrupt, and unpredictable reconfiguration of the problem (Metcalfe and Wiebe, 1987; Sternberg and Davidson, 1995). Because it involves a non-analytical strategy that mobilizes both explicit and implicit processes to produce usually unexpected solutions, insight has been considered a core element of creative problem solving. In many occasions, this has led to culturally equating insight with creativity itself (see Sawyer, 2012 for a critical view).

From Wallas’s four-stage model of preparation, incubation, illumination, and verification (Wallas, 1926, in Hélie and Sun, 2010), to recent work in psychology of insight and intuition, there is a wide agreement that finding creative solutions, despite being usually characterized by a sudden and holistic “Aha!” is not exhausted by this local, more overwhelming aspect of the experience. As mentioned above, the importance of systematic involvement and expertise in the domain of knowledge as conditions that make successful insight-based solutions possible are well known (Runco, 2004; Hennessey and Amabile, 2010; Sawyer, 2012). Likewise, the facilitating effect of incubation periods, where the question is put to rest, has been consistently reported (Sio and Ormerod, 2009; Hélie and Sun, 2010; Baird et al., 2012). Furthermore, it is noteworthy that in many insight-type problem solving tasks, subjects use back-and-forth iterative formulation of possible solutions that are associated with smaller, partial insights (Schooler et al., 1993; Bowden et al., 2005; Hélie and Sun, 2010). These studies show that the time leading up to the moment of insight can be as important as the insight experience itself, by providing the relevant conditions and the (mostly implicit) interpretative context where the Aha!-experience makes sense (see Elements for A Phenomenology of Creative Insight). This is consistent with work in the cognitive neuroscience of insight showing that resting-state brain activity prior to solving a problem can be used to predict whether it will be solved through insight or non-insight strategies (Kounios et al., 2006, 2008). It has also been shown that the likelihood of producing insightful solutions can be modulated by internal states such as mood and attentional distribution (Subramaniam et al., 2009).

As much as the abovementioned approaches have contributed to a more comprehensive understanding of creative insight from process-based psychological and sociological approaches, a descriptive, phenomenological investigation into the way insight is experienced *subjectively* remains surprisingly underdeveloped (Bindeman, 1998; Nelson, 2005). One can only speculate about the reasons for this neglect, some of which are probably related to the underprivileged status first-person data has historically had in the cognitive sciences (Varela and Shear, 1999). However, the tide is slowly turning with mainstream journals publishing studies that take advantage of systematic, rigorous first-person descriptions to guide empirical questions and analysis (Lutz et al., 2002; David et al., 2003; Cosmelli and Thompson, 2007; Christoff et al., 2009). Even if a phenomenological description does not manage, eventually, to bridge the biological, behavioral, and psychological perspectives (Petitot et al., 2000; Schwartz and Metcalfe, 2011), it could provide relevant analogies or heuristics to expand our understanding of this deeply significant experience (Sass, 2001).

## ELEMENTS FOR A PHENOMENOLOGY OF CREATIVE INSIGHT

Consider again how the sudden “Aha!” is experienced during creative insight. Among other aspects, this moment is usually accompanied by a positive affective feeling of something “coming together,” “making sense,” or somehow “falling into place” (see also Schooler et al., 1995, pp. 578–579). From a cognitive psychology approach, this feeling has been proposed to be dependent on a sudden gain in processing fluency (Topolinski and Reber, 2010). From a phenomenological perspective, this moment reveals two complementary aspects of how the experience unfolds in time.

On the one hand, the felt relevance of an answer obtained through insight (independent of its eventual correctness) is always related in a co-generative manner to a prior “wanting” or “lacking” context to which such answer responds. Accordingly, insight solutions are commonly experienced as “gap-filling” (Gruber, 1995; see also Pelaprat and Cole, 2011 for a convergent view regarding imagination), something that can only make experiential sense if both ends of the gap are available at some point of the process (Hélie and Sun, 2010). By virtue of this gap filling, the moment of insight brings with it a very strong and sharp reference to what was going on the moment before. In Runco’s words, “A creative insight is not a quick “aha!” but instead is protracted” (Runco, 2004, p. 662). In this sense, the moment of insight bears a notable resemblance to the resolution of the tip-of-the-tongue experience. It has been proposed that in such cases, this could be associated with succeeding in bringing forth phonological information to match a context of previously activated but incomplete semantic information (Gollan and Brown, 2006). During linear, incremental reasoning the prior context is transparent and fully available in a manner that does not depend on subsequent steps in the thought process. In contrast, when the solution emerges during insight, it retrospectively illuminates the previously opaque context, and makes sense by referring to something that was, until a moment ago, unavailable. In doing so it highlights how the present and the immediate-past are deeply intertwined in the formation of novel meaning.

On the other hand, when the experience of creative insight happens it does not only solve or close a previously posed problem. Both in controlled problem solving settings and during spontaneous insights (those unrelated to a specific contextual problem), it is important to differentiate another aspect of its temporality, one that has received much less attention, especially from the cognitive neurosciences. Insight solutions are creative not only because they solve a given problem in an unexpected and novel way. They are also creative because they can (and usually do) involve a change in the perception or representation of the problem itself (also known as restructuring, see Weisberg, 1995; Chi, 1997). As such, creative insight can open up a potential set of new problems by changing the way the current problem is interpreted vis-à-vis its future consequences.

In contrast to the gap-filling, past-oriented side, this future-oriented aspect is most clearly illustrated through biographical accounts of spontaneous insight, and interviews with individuals—in many cases famous scientists—that discover a new way of looking at an old problem or produce a theoretical synthesis of previously unrelated phenomena, (Csikszentmihalyi and Sawyer, 1995; Gruber, 1995). Poincaré’s description of the consequences of intuiting the order revealed by a mathematical demonstration is illustrative: “A mathematical demonstration is not a simple juxtaposition of syllogisms, it is syllogisms *placed in a certain order*, and the order in which these elements are placed is much more important than the elements themselves. If I have the feeling, the intuition, so to speak, of this order, so as to perceive at a glance the reasoning as a whole, I need no longer fear lest I forget one of the elements, for each of them will take its allotted place in the array, and that without any effort of memory on my part” (Poincaré, 1910, p. 324). And as he goes on to say when speaking about individual differences in mathematical ability: “Others, finally, will possess in a less or greater degree the special intuition referred to, and then not only can they understand mathematics even if their memory is nothing extraordinary, but they may become creators and try to invent with more or less success according as this intuition is more or less developed in them.” Phenomenologically speaking, more than the knowledge just gained, this generative, forward-looking aspect of the experience emphasizes the direction (or rather potential directions) toward which one is left facing, so to speak, as a consequence of one’s insight into the problem’s nature. During spontaneous insight this aspect of creative insight can be very powerful as one’s flow of experience becomes unexpectedly diverted toward the consequences of the realization.

It is worth considering that the two-sided past-closing/future-opening structure is analogous across other levels of organization. For instance, it is consistent with the view of the brain and body as a system driven mainly by endogenous, historically dependent dynamics, which support action perception cycles by continuously minimizing prediction errors (Friston, 2005; Clark, 2013). As pointed out by neurodynamicists reaching back to Karl Lashley, it makes no sense to analyze neural activity purely as happening instantaneously or just as a reaction to impinging stimuli, while ignoring its ongoing, predictive nature (Freeman, 2000; Thompson and Varela, 2001; Raichle and Gusnard, 2005; Cosmelli et al., 2007). In other words, ongoing brain activity creatively prefigures virtual, motor and perceptual possibilities by continuously bringing forth our history of interactions into the present “now” (Varela, 2000). Or consider the dynamics of biological evolution, whose similarities with creativity and insight have been pointed out previously (Simonton, 1999, 2013). For example, in an analogous way to what happens during restructuring, it can be argued that, evolutionarily, flying is not just a prior problem waiting to be solved with wings or membranous forelimbs (as in bats, see Sears et al., 2006). Flying is also possible *as a set of new problems* because wings or membranous forelimbs are available. Evolution is not exhausted by pure random variation; it is the emergence of novelty within boundary conditions, conditions that are (self) affected precisely by that which emerges. It is tempting to think that such analogies might point to underlying common mechanisms in biological systems (see also Perkins, 1995).

## CONSEQUENCES AND FUTURE DIRECTIONS

We believe that the previous analysis has a number of consequences that are relevant for studies on creative insight, but also for a phenomenology of the flow of experience. Take for instance the past-oriented, gap-filling aspect. As we discussed above, it brings to the fore in a very palpable manner, the intimate relation that exists between the present and the immediate past in experience (James, 2007; first edition-1890). Such past-looking, *retentive* aspect that is always available in the experience of the present “now” has been consistently described in the phenomenological philosophy tradition (Merleau-Ponty, 1976; Husserl, 1991; Sherover, 2001) and has been proposed to play a role in providing unity to the flow of experience (Varela, 2000). The fact that it is made so evident during the “Aha!” moment is what we wish to highlight here: it suggests that spontaneous occurrences of insight situations could be a natural target for the phenomenological inquiry into the ongoing flow of experience. One of the difficulties faced by phenomenological investigation is pinpointing the object of description, especially when its appearance is unpredictable. By providing a recognizable, easily relatable, “anchor point,” creative insight can facilitate taking a phenomenological attitude toward a well-defined target that preserves the spontaneity of the flow of experience. In this sense, it could play a role similar to that proposed by Schwartz and Metcalfe (2011) for tip-of-the-tongue experiences, becoming a natural candidate for phenomenological study that can be contrasted with psychological, cognitive or neuroscientific accounts.

It remains an open question whether a more in-depth exploration of the moments leading up to the experience of sudden insight can be the subject of phenomenological investigation, for the sake of understanding creative insight itself. As studies since Metcalfe and Metcalfe and Wiebe’s (1987) seminal work have shown much of what is going on during the incubation period prior to the moment of the Aha! experience is not accessible to the subject. This would in principle challenge the utility of phenomenological descriptions of this moment of the experience. However, one need not stay exclusively with the phenomenological approach. That resting state activity prior to confronting a problem can, in principle, predict whether a problem will be solved through insight or not suggests a possible strategy. Consider the proliferation of signal analysis algorithms that are currently available for ongoing EEG decomposition in the context of brain–computer interface development (Krusienski et al., 2011). Subjects could be prompted for descriptions of their ongoing experience when characteristic features of brain activity associated with insight solutions (i.e., changes in peak frequency in the low alpha band or drop in alpha band power over mid frontal and left anterior temporal regions, see Kounios et al., 2006) are detected. In particular, the locus of attention could be a relevant target as it has been proposed that a diffuse distribution of visual attention might be a characteristic feature of insight-based solutions (Kounios et al., 2008, p. 283, see also Subramaniam et al., 2009). This would be a brain activity-based sophistication of the strategy used originally by Metcalfe and Wiebe (1987), whereby subjects were asked at different moments prior to the Aha! experience to evaluate their feeling of approaching a solution.

The more future-oriented, *protentive* side of insight, on the other hand, underscores the self-affecting nature of experience in a very clear way. Restructuring implies that the problem takes its final form (meaning, consequences, etc.) only when the solution is discovered. As such, it suggests that the less studied restructuring aspect could be a relevant focus, for example, when seen in a more learning oriented setting. Most psychological and cognitive neuroscience studies have dealt with those mental or brain processes leading up to or facilitating the moment of restructuring (Metcalfe and Wiebe, 1987; Bowden et al., 2005; Kounios et al., 2008; Kounios and Beeman, 2009; Subramaniam et al., 2009; Eubanks et al., 2010). However, much less is known regarding the consequences of restructuring for future, possibly recurrent encounters with similar problems. For instance, it would be interesting to study if and under which conditions repetitive exposure to certain type of insight problems can eventually lead to generalizations or the development of strategies to deal with them (see also Weisberg, 1995 and Eubanks et al., 2010). If this restructuring aspect, which is more clearly available in understanding-type insights (Gruber, 1995), is characteristic of creative insight in general, one would predict that every instance of closed-problem insightful resolution would change to some degree the way the problem is judged. Such change might be small and difficult to detect for each individual event, but if it exists, it would be reasonable to expect that at some point of recurrent encounter with a given type of such problems, some kind of meta-insight regarding the underlying logic should become available to the person. This expertise-related prediction should, in principle, be amenable to experimental testing with current problem-solving based approaches both in terms of changes in behavior and in brain activity.

An obvious limitation of any phenomenological enterprise is that it deals, by definition, with experience as described by the same subject of that experience. Here we have taken this as a starting point but we have strived to triangulate our observations with psychological studies and cognitive neuroscience results in the spirit of cross-validation. We believe that the results of this triangulation are encouraging and point to potentially relevant lines for further inquiry. In the limited space of this essay we cannot tackle a full-fledged phenomenological investigation into creative insight. We have, however, focused on a two outstanding aspects that warrant further attention not only from a phenomenological perspective but also in terms of psychological and biological approaches. These descriptions are open to contrast with other researcher’s experience on the one hand, and with future results from experimental approaches on the other. By its very nature, phenomenological investigation has to start from the individual and seek intersubjective contrast, refinement, and eventually, validation. The perspective here adopted aims to invite others to adopt a much-needed phenomenological stance in order to contribute to the understanding of the experience of creative insight.

We have argued here that creativity in general—and insight in particular—offers an extremely rich case, phenomenologically speaking, for the study of the temporal structure of human experience, one that represents a challenging venue of research. Indeed, creative insight brings forth and coherently embodies the future-past polarity of experience in a very explicit way. As such, it is at the same time the experience of something surprisingly novel but profoundly familiar.

## Conflict of Interest Statement

The authors declare that the research was conducted in the absence of any commercial or financial relationships that could be construed as a potential conflict of interest.
